# Genetic Predictors of Curve Progression and Brace Treatment Response in Adolescent Idiopathic Scoliosis: Toward Personalized Management

**DOI:** 10.3390/diagnostics16203330

**Published:** 2026-10-14

**Authors:** Alexandru Ulici, Alexandru Ciobanu, Alexandru Herdea

**Affiliations:** 1 11th Department of Pediatric Orthopedics, Carol Davila University of Medicine and Pharmacy, Bd. Eroii Sanitari Nr. 8, 050474 Bucharest, Romania; alexandru.ulici@umfcd.ro (A.U.); alexandru.herdea@umfcd.ro (A.H.); 2 Pediatric Orthopedics Department, Grigore Alexandrescu Children’s Emergency Hospital, 011743 Bucharest, Romania

**Keywords:** adolescent idiopathic scoliosis, genetics, curve progression, brace treatment, treatment response, genetic predictors, polygenic risk, personalized medicine

## Abstract

Adolescent idiopathic scoliosis (AIS) is characterized by substantial variability in curve progression and response to conservative treatment, making accurate prognostic assessment a major clinical challenge. Although established clinical and radiographic factors contribute to risk stratification, they do not fully explain individual differences in disease evolution or brace treatment outcome. This review critically examines current evidence on genetic determinants of curve progression and brace treatment response in AIS, with particular emphasis on their potential contribution to individualized risk prediction. Candidate-gene studies have reported associations involving hormonal, metabolic, developmental, and skeletal pathways, while studies of brace response have identified potential predictive associations involving *ERα*, *TPH1*, *VDR*, *VDBP*, and *BNC2*. Multigene approaches, including ScoliScore, have further explored the potential of polygenic information for prognostic assessment. However, reported genetic effects are generally modest, replication across populations remains limited, and substantial heterogeneity exists in study design, outcome definitions, and characterization of brace treatment. Importantly, current evidence remains insufficient to demonstrate clinically meaningful predictive value of genetic information beyond established clinical factors. Future research should prioritize large prospective multicenter cohorts, standardized definitions of progression and treatment outcome, objective assessment of brace adherence and correction, progression-specific polygenic approaches, and external validation across diverse populations. Ultimately, integrating genetic information with clinical, radiographic, developmental, biomechanical, and treatment-related variables may provide a more comprehensive framework for individualized prediction and precision management of AIS.

## 1. Introduction

Adolescent idiopathic scoliosis (AIS) is characterized by substantial variability in its clinical course. While some spinal curves remain relatively stable throughout growth, others demonstrate progressive deformity and may ultimately reach magnitudes requiring surgical treatment. Accurate identification of patients at increased risk of curve progression therefore represents a major challenge in AIS management, particularly during periods of rapid skeletal growth. Current clinical assessment relies primarily on parameters such as age, skeletal maturity, baseline Cobb angle, curve pattern, and remaining growth potential. Although these factors provide valuable prognostic information, considerable interindividual variability remains, suggesting that additional biological determinants may influence disease evolution [1,2].

Familial aggregation, twin studies, and genome-wide association studies have established a substantial genetic contribution to AIS [3,4,5]. However, most genetic studies have focused on susceptibility to disease rather than on the subsequent clinical behavior of an established spinal deformity. The distinction between genetic susceptibility and genetic determinants of progression is clinically important. Variants that influence the development of AIS may not necessarily determine whether an existing curve will remain stable or progress, and progression-associated loci may act through biological pathways related to skeletal growth, hormonal regulation, connective-tissue properties, neuromuscular development, and epigenetic regulation [4,6].

The potential contribution of genetic factors becomes particularly relevant in patients undergoing conservative treatment. Bracing remains a cornerstone of treatment for skeletally immature patients with progressive AIS, while physiotherapeutic scoliosis-specific exercises, particularly the Schroth Method, may provide additional benefits as part of conservative management [7,8]. In patients with severe or rapidly progressive AIS who ultimately require surgical correction, preoperative strategies such as halo-gravity traction may also be used to reduce curve magnitude before definitive surgery [9]. Treatment outcomes vary considerably among individuals. Brace effectiveness is influenced by several established factors, including initial curve magnitude, skeletal maturity, curve characteristics, in-brace correction, and treatment adherence [7,10]. Nevertheless, patients with apparently similar clinical characteristics and treatment exposure may experience different outcomes, raising the possibility that genetic variation may contribute to individual differences in treatment response.

Candidate-gene studies have investigated polymorphisms involving estrogen signaling, serotonin metabolism, vitamin D signaling, growth regulation, and other biological pathways as potential predictors of curve behavior or brace treatment response [6,10]. More recently, genome-wide association studies, progression-associated variants, epigenetic biomarkers, and polygenic approaches have expanded this concept beyond individual candidate polymorphisms [4,5,11,12]. Genetic prognostic tests, including ScoliScore, have further illustrated the possibility of combining information from multiple genetic markers to estimate clinical risk [13]. However, inconsistent replication, population-specific associations, heterogeneous definitions of progression and treatment failure, and limited external validation have restricted the translation of these findings into routine clinical practice [6,10].

This review aims to critically evaluate the current evidence regarding genetic determinants of curve progression and brace treatment response in adolescent idiopathic scoliosis. Particular attention is given to the transition from individual candidate-gene associations to multigenic, polygenic, epigenetic, and integrated predictive approaches, the methodological limitations of existing studies, and the potential role of genetic information in future personalized management strategies for AIS.

## 2. Literature Search and Evidence Selection

A structured literature search was conducted in PubMed/MEDLINE, Scopus, and Web of Science to identify studies evaluating genetic or epigenetic factors associated with curve progression, curve severity, or brace-treatment outcomes in adolescent idiopathic scoliosis (AIS). The search covered publications from database inception through September 2026. Particular attention was given to studies published during the most recent 10-year period (2016–2026) to ensure that contemporary evidence, including recent genome-wide, epigenetic, polygenic, and treatment-response studies, was adequately represented. Earlier landmark studies were retained when directly relevant to the development or validation of genetic prognostic approaches.

Search terms included combinations of “adolescent idiopathic scoliosis”, “AIS”, “genetic”, “genetics”, “polymorphism”, “SNP”, “genome-wide association”, “GWAS”, “polygenic risk score”, “curve progression”, “curve severity”, “brace”, “bracing”, “brace response”, and “treatment response”. Reference lists of relevant reviews and eligible primary studies were additionally screened to identify potentially relevant publications.

Studies were considered eligible when they evaluated a genetic or epigenetic marker in relation to curve progression, curve severity, or brace-treatment outcome in patients with AIS. Studies addressing AIS susceptibility alone were not considered evidence of progression or treatment response unless the investigated marker was subsequently evaluated in relation to one of these outcomes. Because cross-sectional curve severity and longitudinal curve progression represent distinct phenotypes, these outcomes were considered separately throughout the review. Primary studies were prioritized for the evidence tables, while systematic reviews and meta-analyses were used to contextualize the findings, identify additional eligible primary studies, and summarize previously synthesized evidence where appropriate.

Given the substantial heterogeneity in study design, genetic markers, populations, phenotype definitions, and treatment-related outcomes, the available studies were interpreted according to study design, sample size, phenotype definition, replication status, and external validation. Large genome-wide studies and independently replicated findings were considered to provide stronger evidence than small exploratory candidate-gene studies. Small studies were retained when they directly addressed progression or brace-treatment response because of the limited literature specifically addressing these outcomes; however, their exploratory nature and limited generalizability are explicitly identified in the text and summary tables.

Where at least two studies provided sufficiently comparable quantitative data for the same genetic variant and phenotype, an exploratory marker-specific meta-analysis was performed. Random-effects models were fitted using restricted maximum likelihood (REML) estimation, with Hartung–Knapp adjustment for uncertainty in the pooled effect. For dichotomous outcomes, effect estimates were expressed as odds ratios (ORs) with 95% confidence intervals (CIs), whereas continuous Cobb-angle outcomes were synthesized as mean differences (MDs) in degrees. Statistical heterogeneity was assessed using the I^2^ statistic and Cochran’s Q test. Because only two or three studies were available for the marker-specific analyses, funnel plots were generated for descriptive visualization only and were not used to draw conclusions regarding publication bias.

The meta-analyses and corresponding forest and funnel plots were generated using the Numiqo online meta-analysis platform.

## 3. Genetic Determinants of Curve Progression

The genetic architecture underlying curve progression in adolescent idiopathic scoliosis remains substantially less defined than the genetic determinants of disease susceptibility. Large genome-wide association studies have identified multiple loci associated with AIS susceptibility across different populations [4,5,14]. However, most of these investigations used disease occurrence rather than longitudinal curve progression as their primary phenotype. Consequently, a susceptibility-associated variant should not automatically be interpreted as a marker of progression once scoliosis has developed [6].

Several candidate-gene and genome-wide studies have nevertheless reported associations between genetic variation and curve magnitude, severity, or progression. The implicated genes span heterogeneous biological pathways, including hormonal signaling, skeletal growth, extracellular-matrix regulation, neurotransmitter metabolism, and developmental processes [4,6]. More recent genome-wide studies have expanded the spectrum of AIS susceptibility loci [15], while progression-focused studies have begun to identify loci whose effects appear more closely related to curve behavior than to disease occurrence itself [16], supporting the concept that susceptibility and progression may have partially distinct genetic architectures.

For the purposes of this review, cross-sectional curve severity and longitudinal curve progression are considered distinct phenotypes. Cross-sectional severity describes curve magnitude measured at a particular time point or comparisons between severity-defined groups, whereas longitudinal progression requires documented change in curve magnitude over time. Associations derived exclusively from cross-sectional severity studies are therefore not interpreted as direct evidence of prognostic ability for future curve progression.

### 3.1. Early Candidate-Gene Studies

Growth-related pathways represent biologically plausible modifiers of curve behavior because the period of greatest risk for AIS progression overlaps with adolescent skeletal maturation. Genetic studies involving the growth hormone–IGF1 axis have therefore investigated whether variation in these pathways may influence curve severity. Moon et al. reported associations involving IGF1 and LAPTM4B in a Korean AIS population, contributing to the hypothesis that growth-related genetic variation may influence the clinical phenotype of established scoliosis [17]. Nevertheless, the relatively small sample size and population-specific nature of this study limit the generalizability of these findings, and IGF1 cannot currently be regarded as an independently validated predictor of curve progression [4,6,17].

Metabolic pathways have also emerged as potential modifiers of AIS progression. Wright et al. investigated the combined effects of functional variants in COMT and MTHFR in patients with AIS [18]. COMT is involved in catecholamine metabolism, whereas MTHFR participates in folate and homocysteine metabolism, providing biologically distinct pathways through which genetic variation could potentially influence skeletal or neuromuscular processes.

The study included 72 patients with longitudinal Cobb-angle measurements and qualifying COMT and MTHFR genotypes. Progression, defined as an increase in Cobb angle of at least 20° or progression to spinal surgery between diagnosis and skeletal maturity, occurred in 78.0% of patients carrying combined low-activity genotypes compared with 48.4% of the comparison group [18]. Although these findings support the investigation of combined genetic effects, the small cohort, retrospective design, selected genotype groups, and absence of external validation substantially limit immediate clinical interpretation. The results should therefore be considered hypothesis-generating rather than evidence supporting routine COMT/MTHFR genotyping.

### 3.2. Hormonal and Metabolic Modifiers of Progression

Hormonal regulation has long been investigated as a potential contributor to variability in AIS severity, particularly because clinically significant progression frequently occurs during puberty. Estrogen receptor polymorphisms are among the most extensively investigated candidate variants in this context. Associations involving ESR1 and ESR2 have been reported for curve severity or progression in several populations, although results have not been uniform [4,11]. A recent systematic review and meta-analysis found evidence for associations between selected estrogen receptor polymorphisms and progressive phenotypes, while not supporting these variants as consistent determinants of AIS occurrence [11]. Estrogen-related genetic variation may therefore be more appropriately considered a potential modifier of clinical phenotype than a primary susceptibility mechanism.

Leptin signaling represents another biologically plausible pathway because of its relationship with energy metabolism, puberty, bone homeostasis, and skeletal growth. Genetic variation involving LEP and LEPR has been investigated in AIS, but current evidence more strongly supports associations with susceptibility or metabolic phenotype than a reproducible independent effect on longitudinal curve progression [4,6]. Accordingly, these variants should not presently be considered established progression biomarkers.

Epigenetic regulation provides an additional mechanism through which genetic background and developmental or environmental influences may interact. Meng et al. compared genome and methylome profiles in monozygotic twins discordant for curve progression and subsequently validated candidate methylation sites in independent samples [12]. Hypomethylation of cg01374129 was independently associated with curve progression and differentiated progressive from non-progressive cases with an AUC of 0.827, although further external validation is required before clinical implementation [12].

Additional evidence has implicated the methylation of genes involved in skeletal and developmental pathways. Increased COMP promoter methylation was associated with lower COMP expression and greater Cobb-angle magnitude, while methylation changes involving PITX1 have also been associated with curve magnitude [19,20]. These findings support epigenetic biomarkers as a potentially important complement to germline genetic markers, but their predictive value remains insufficiently validated for routine clinical use.

### 3.3. From Susceptibility Loci to Progression-Associated Variants

One of the principal limitations of AIS genetics is that the rapid expansion of susceptibility loci identified through GWAS has not been accompanied by an equivalent expansion of validated progression markers. Variants near LBX1, ADGRG6/GPR126, BNC2, and other loci have repeatedly been associated with AIS susceptibility across populations [5,15]. However, association with disease occurrence does not necessarily imply an effect on subsequent curve behavior [6].

An important progression-specific finding emerged from a genome-wide association and replication study involving 2543 patients with AIS, which identified a locus at chromosome 11q14.1 associated with curve progression. Functional analyses implicated rs35333564 in MIR4300HG, with the risk allele reducing enhancer activity [16]. Subsequent investigation in a Chinese population replicated the association of rs35333564 with curve severity and linked MIR4300 expression with curve magnitude, providing cross-population support for this locus as a potential progression modifier [21].

BNC2 represents another locus of particular interest. Following its identification as an AIS susceptibility locus and functional evidence supporting its biological relevance, associations have been replicated across populations [22,23]. More recently, BNC2 variation has also been investigated in relation to brace treatment response, further illustrating how susceptibility-associated loci may potentially influence clinically relevant phenotypes after disease onset [24]. Nevertheless, these observations require independent validation before BNC2 can be incorporated into prognostic algorithms.

Very recent genome-wide evidence has further expanded the spectrum of potential severity-associated loci. A 2026 GWAS comparing female patients with severe curves (>40°) and mild curves (<30°) identified and replicated associations involving GAK rs2061846 and KLF12 rs7330031. The GAK risk allele was associated with an odds ratio of 1.32 for severe curves, while the KLF12 risk allele had an odds ratio of 1.46; KLF12 expression in paraspinal muscle was also negatively correlated with Cobb angle [25]. These results are promising but should be interpreted primarily as severity-associated rather than definitive longitudinal progression markers because the phenotype was based on a comparison of severe and mild curves rather than prospective observation of curve progression.

This distinction has important implications for future study design. Progression-focused genetic studies should prioritize longitudinal phenotyping and standardized outcomes based on change in Cobb angle, progression beyond clinically meaningful thresholds, or progression to surgery. They should also account for age, sex, skeletal maturity, baseline curve magnitude, treatment exposure, brace adherence, and duration of follow-up. Such designs would improve the ability to distinguish genetic determinants of disease initiation from those influencing subsequent clinical evolution.

### 3.4. Quantitative Synthesis of Selected Replicated Variants

**Figure 1:**
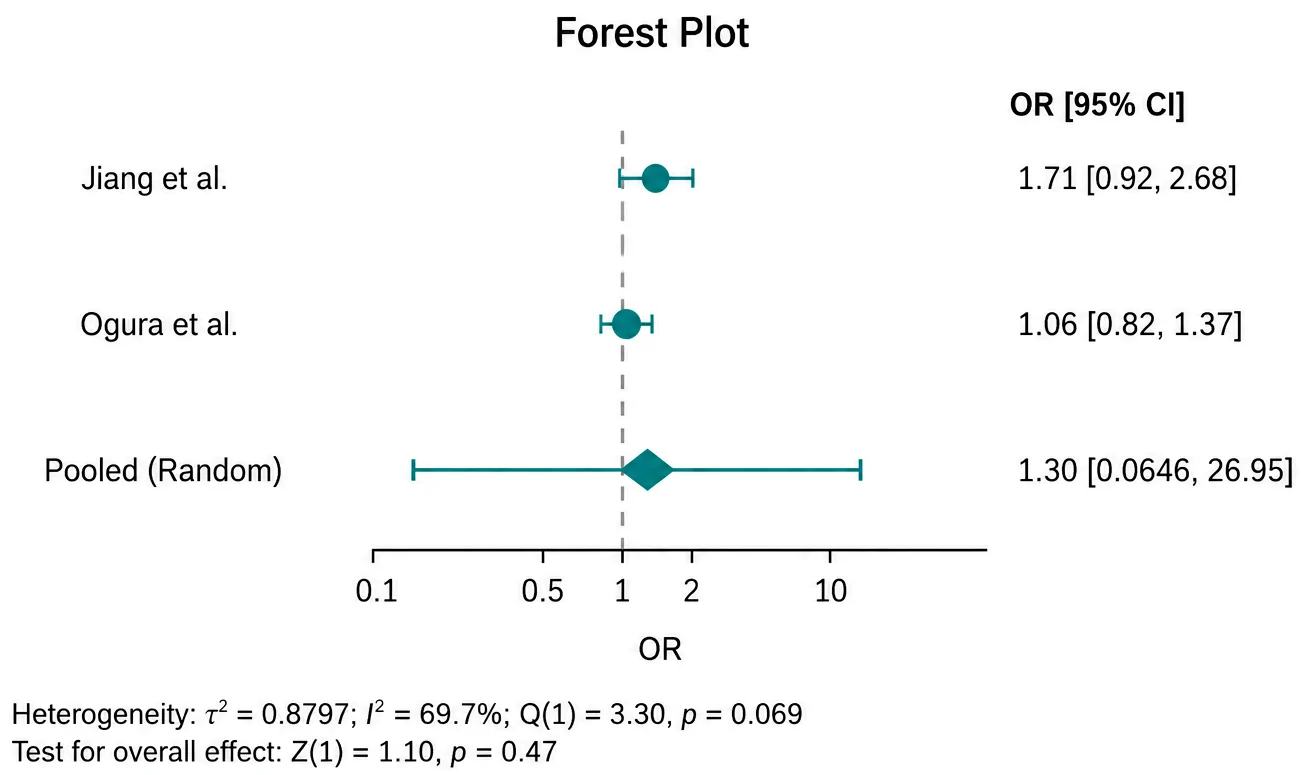
Random-effects meta-analysis of TIMP2 rs8179090 and AIS curve phenotype. Individual and pooled odds ratios are presented with 95% confidence intervals. The pooled estimate was calculated using REML with Hartung-Knapp adjustment. The studies included in the meta-analysis were Jiang et al. [26] and Ogura et al. [27].

**Figure 2:**
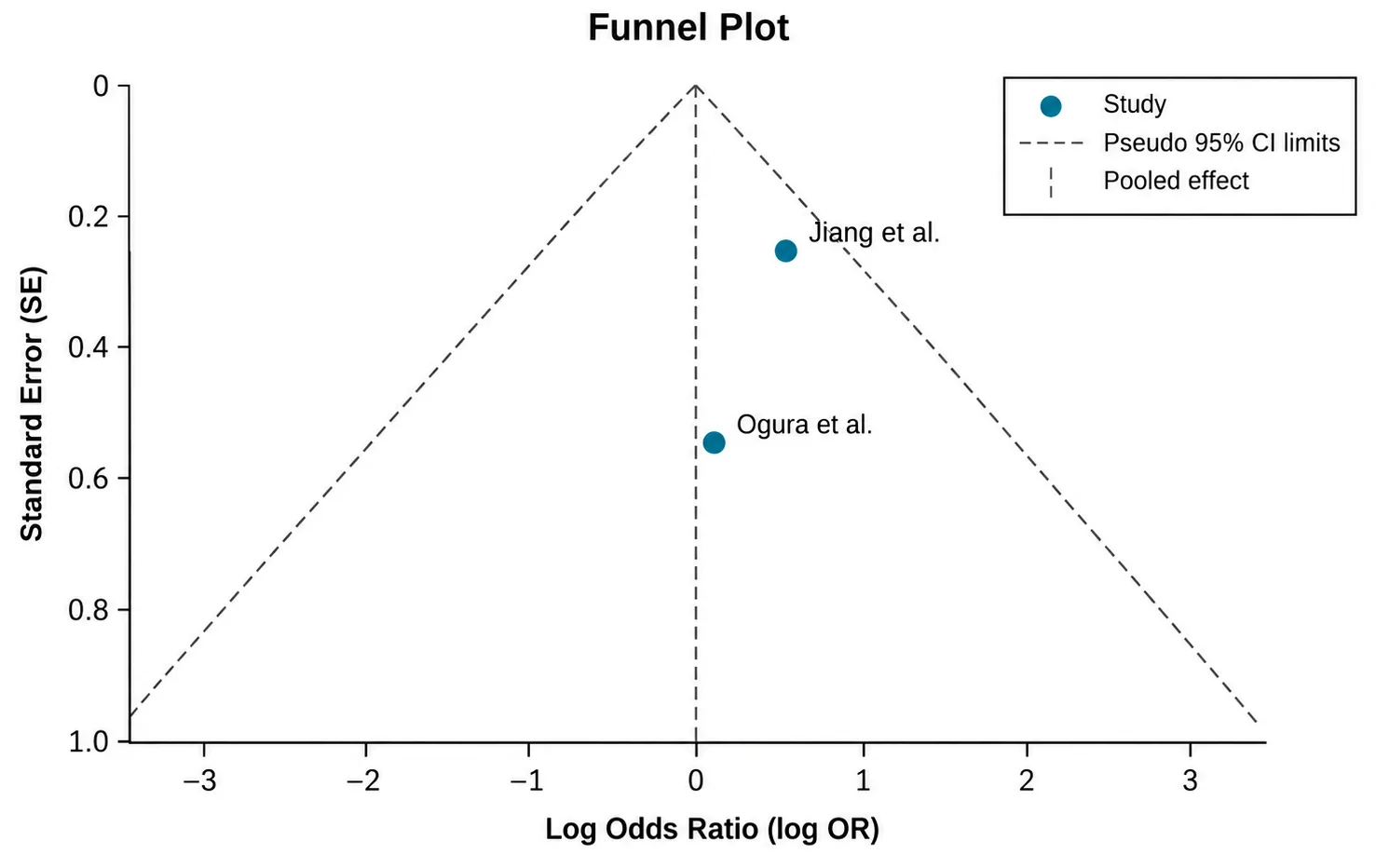
Funnel plot for the meta-analysis of TIMP2 rs8179090 and AIS curve phenotype. Due to the small number of included studies (*n* = 2), this plot should be interpreted descriptively only [26,27].

Quantitative synthesis was feasible for TIMP2 rs8179090 based on two studies reporting compatible effect estimates [26,27]. The individual odds ratios were 1.71 (95% CI 1.09–2.68) and 1.06 (95% CI 0.82–1.36). Under a random-effects model using REML estimation and Hartung–Knapp adjustment, the pooled OR was 1.30 (95% CI 0.06–26.05; *p* = 0.47), with substantial between-study heterogeneity (I^2^ = 69.7%; Q = 3.30, *p* = 0.069). Thus, the initially reported association was not supported by a statistically significant pooled effect, and the very wide confidence interval reflects the substantial uncertainty resulting from the availability of only two studies (Figure 1). The corresponding funnel plot is presented in Figure 2 for descriptive purposes only, given the inclusion of just two studies.

**Figure 3:**
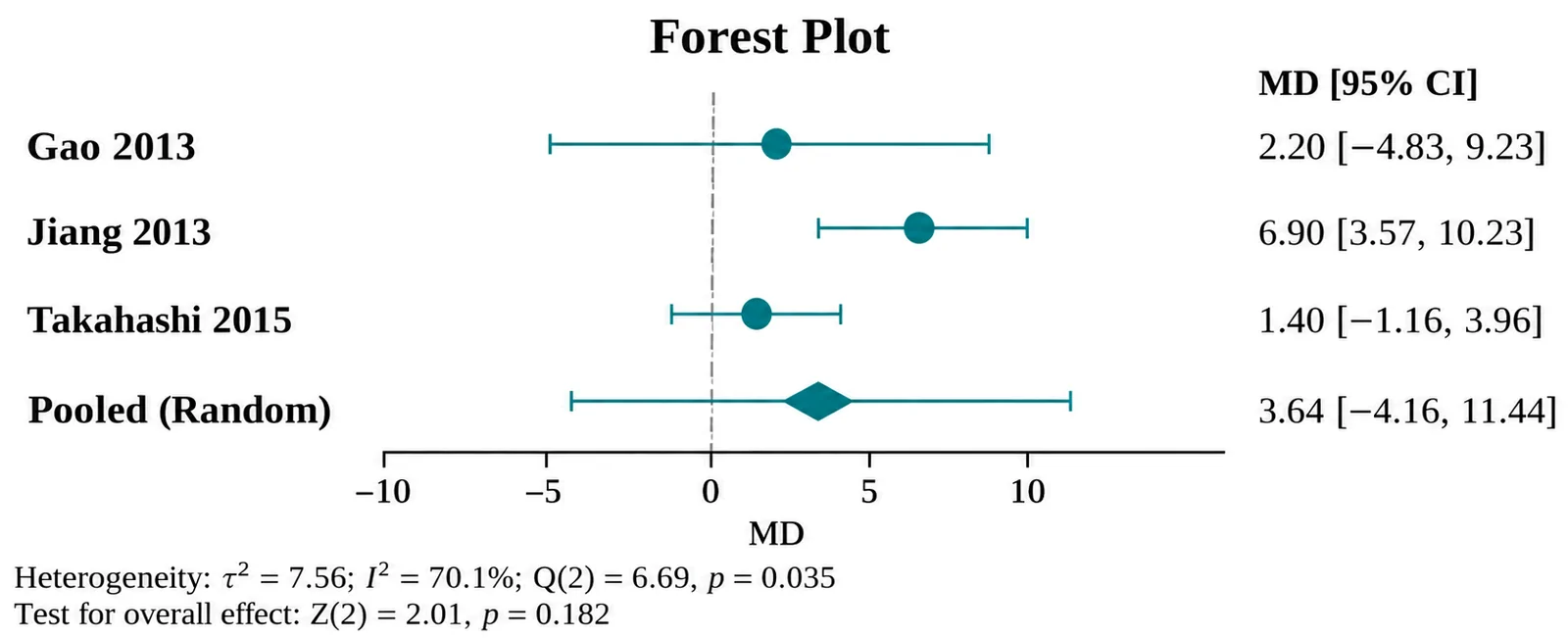
Random-effects meta-analysis of LBX1 rs11190870 and AIS curve severity. Individual and pooled meand differences (MD) are presented with 95% confidence intervals. The pooled estimate was calculated using REML with Hartung-Knapp adjustment. The studies included in the meta-analysis were Gao et al. [28], Jiang et al. [29], and Takahashi et al. [30].

**Figure 4:**
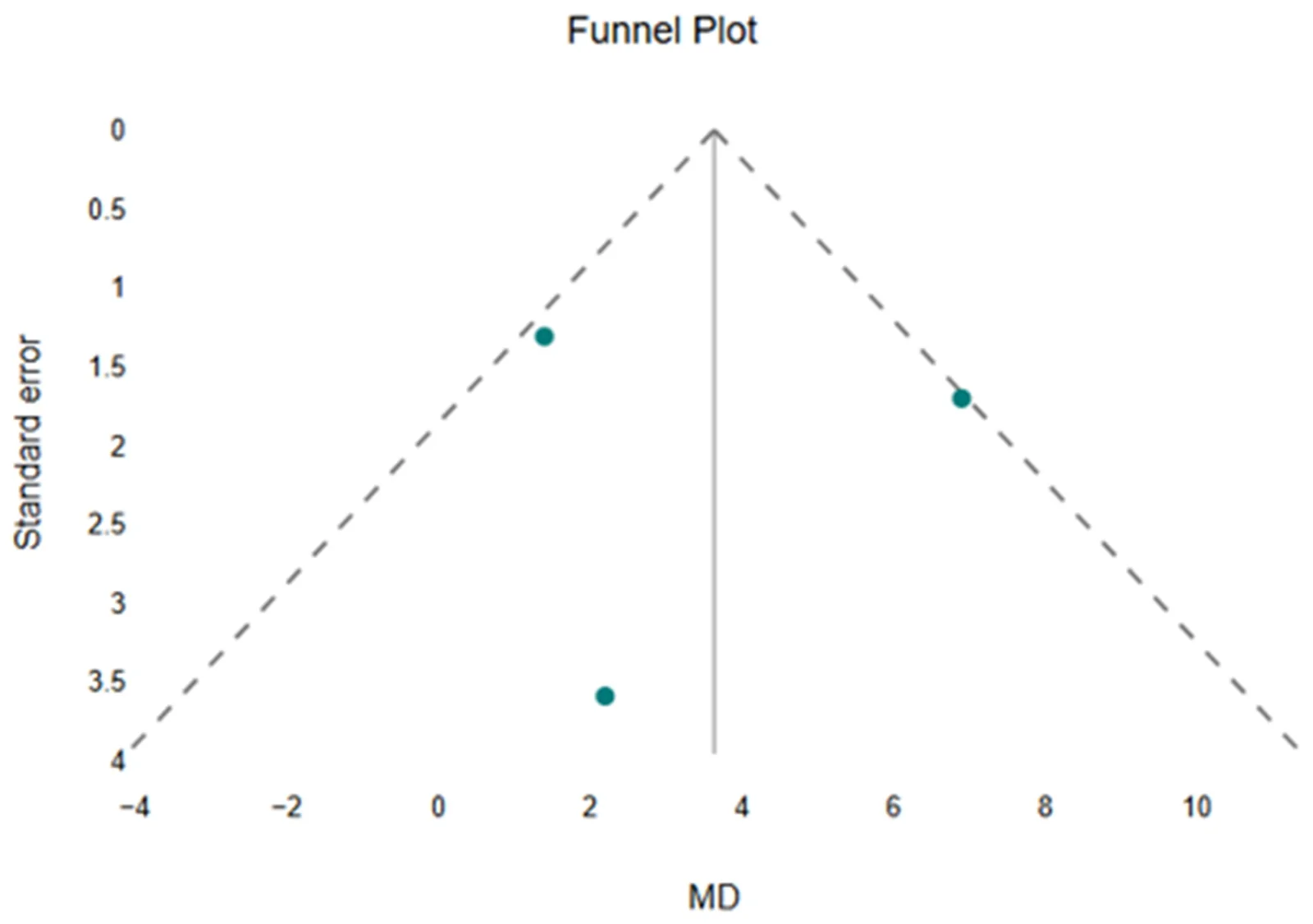
Funnel plot for studies evaluating LBX1 rs11190870 and curve severity. Because only three studies were included, the plot is presented descriptively and does not permit a reliable assessment of publication bias.

A second quantitative synthesis evaluated the association between LBX1 rs11190870 and curve severity using genotype-specific Cobb-angle data from three studies [28,29,30]. Comparing the TT and CC genotypes, the individual mean differences were 2.20° (95% CI −4.83 to 9.23), 6.90° (95% CI 3.57–10.23), and 1.40° (95% CI −1.16 to 3.96). The random-effects pooled mean difference was 3.64° (95% CI −4.16 to 11.44; *p* = 0.182), with substantial heterogeneity (I^2^ = 70.1%; Q = 6.69, *p* = 0.035). Therefore, although one individual study reported a significant association, the pooled estimate did not demonstrate a statistically significant difference in Cobb-angle severity between the TT and CC genotypes. Importantly, this analysis addresses cross-sectional curve severity rather than prospective longitudinal progression (Figure 3). The corresponding funnel plot is presented in Figure 4 for descriptive purposes only, given the inclusion of just three studies.

No additional quantitative pooling was performed because the remaining studies evaluated different genetic variants, genetic models, or non-comparable progression and treatment-response phenotypes.

### 3.5. Current Limitations of Genetic Progression Markers

Despite the growing number of reported associations, no individual variant or currently available genetic panel can be considered an established standalone predictor of AIS curve progression [4,6]. Replication across populations remains problematic, while differences in ancestry, sample size, phenotype definition, skeletal maturity, treatment exposure, and statistical methodology contribute substantially to inconsistent findings.

The exploratory quantitative syntheses performed in the present review further illustrate these limitations. For TIMP2 rs8179090, the pooled effect did not demonstrate a statistically significant association, despite a positive result in one of the individual studies, and the estimate was characterized by substantial imprecision and between-study heterogeneity. Similarly, for LBX1 rs11190870, the pooled difference in Cobb-angle severity between the TT and CC genotypes was not statistically significant and showed substantial heterogeneity across studies. These findings emphasize the importance of independent replication and quantitative synthesis before individual genetic associations are interpreted as clinically relevant prognostic markers. They also highlight the need to distinguish cross-sectional associations with curve severity, as evaluated in the LBX1 analysis, from the true longitudinal prediction of curve progression.

Interpretation of the candidate-gene literature is further complicated by selective emphasis on positive associations. Non-significant findings and unsuccessful replication attempts are equally important for evaluating the credibility of proposed genetic markers, particularly when initial associations arise from small cohorts or multiple candidate variants are tested. Accordingly, the present review considers both positive and negative or non-replicated findings when such evidence is available. Failure to reproduce an initial association in an independent population substantially weakens its potential prognostic relevance, even when the original biological hypothesis remains plausible. Consequently, isolated candidate–gene associations should be regarded primarily as hypothesis-generating unless supported by independent replication, whereas genome-wide findings with replication provide a comparatively stronger level of evidence. Another limitation is the historical emphasis on candidate-gene approaches. Although biologically plausible, such studies are particularly vulnerable to small sample sizes, population stratification, multiple-testing effects, and publication bias [6]. Genome-wide approaches, epigenetic biomarkers, and polygenic models offer broader strategies, but these approaches also require large cohorts, standardized phenotypes, independent replication, and external validation.

Overall, the current evidence supports a genetic and epigenetic contribution to AIS curve behavior but does not yet provide a sufficiently robust or generalizable standalone marker for clinical prediction. The most promising direction is therefore likely to involve multivariable models integrating genetic and epigenetic information with established clinical, radiographic, developmental, and treatment-related determinants rather than attempting to replace conventional prognostic assessment with isolated molecular markers.

The main genetic variants investigated in relation to AIS curve progression are summarized in Table 1.

## 4. Genetics of Brace Treatment Response

### 4.1. Candidate Genetic Markers of Brace Response

Brace treatment represents the primary non-operative intervention for skeletally immature patients with AIS who are considered at risk of curve progression. Despite its established effectiveness, substantial variability in treatment outcome is observed among patients. Brace success is influenced by several clinical and treatment-related factors, including initial curve magnitude, skeletal maturity, curve pattern, in-brace correction, and adherence [10,31,32,33]. However, differences in outcome among patients with apparently comparable clinical characteristics and treatment exposure have prompted investigation into whether genetic variation may also contribute to brace responsiveness.

One of the earliest studies specifically investigating genetic predictors of brace treatment outcome was conducted by Xu et al. [34]. The authors evaluated 312 patients with AIS treated with bracing and investigated polymorphisms in genes previously implicated in AIS susceptibility or curve severity, including estrogen receptor alpha (ERα), estrogen receptor beta (ERβ), tryptophan hydroxylase 1 (TPH1), melatonin receptor 1B (MTNR1B), and matrilin-1 (MATN1). Brace failure was defined as curve progression of more than 5° relative to the baseline or progression requiring surgical intervention.

Brace failure occurred in 90 out of 312 patients (28.8%). The ERα rs9340799 G allele and TPH1 rs10488682 A allele were more frequent among patients with unsuccessful brace treatment, and both ERα and TPH1 remained independent predictors in logistic regression [34]. These findings provided early evidence that genetic variation might contribute to brace response. Nevertheless, the candidate-gene design, population specificity, and absence of extensive external validation limit the direct clinical applicability of these markers.

### 4.2. Vitamin D-Related Genetic Variation

Vitamin D-related pathways have also been investigated in relation to brace treatment response. Wang et al. examined polymorphisms in the vitamin D receptor (VDR) and vitamin D-binding protein (VDBP) genes in patients with AIS, assessing their associations with both disease susceptibility and brace effectiveness [35]. The study identified higher frequencies of the VDR BsmI Bb genotype and B allele, as well as the VDBP rs222020 CC genotype and C allele, among patients with AIS compared with controls [35].

Importantly, genetic variation was also associated with differences in response to brace treatment. Patients carrying the VDR BsmI Bb genotype or VDBP rs222020 CC genotype demonstrated lower correction rates for several radiographic parameters following treatment, including Cobb angle and measures of vertebral translation and rotation [35].

However, these associations remain insufficient for clinical application. The study represents evidence from a specific population and has not established that VDR or VDBP genotyping provides predictive information beyond established clinical factors. Independent replication and prospective validation are therefore required before vitamin D-related polymorphisms can be incorporated into treatment-selection algorithms.

### 4.3. Progression-Associated Variants as Predictors of Brace Failure

A more recent strategy has been to investigate whether genetic variants previously associated with AIS progression can also predict the failure of brace treatment. This approach differs conceptually from earlier candidate-gene studies because candidate variants are selected on the basis of evidence linking them to disease progression rather than solely to biological plausibility or AIS susceptibility.

Dai et al. evaluated five progression-associated single-nucleotide polymorphisms in a cohort of 259 female patients with AIS undergoing brace treatment [24]. The investigated variants included rs10738445 in BNC2, rs12946942 near SOX9/KCNJ2, rs1978060 in TBX1, rs1017861 in CHD7, and rs35333564 in MIR4300HG. Among these variants, only BNC2 rs10738445 showed a significant association with brace outcome. The C allele was associated with an increased risk of brace failure (OR 1.59), whereas no significant associations were identified for the remaining four SNPs [24].

This finding is of particular interest because BNC2 had previously emerged as an AIS susceptibility locus [22,23]. Functional studies have demonstrated allele-dependent regulation of BNC2 expression, while subsequent investigations have supported its relevance across different AIS populations [22,23]. The association between BNC2 variation and brace outcome therefore provides a potential connection between genetic susceptibility, curve phenotype, and treatment response.

Nevertheless, the modest effect size of the BNC2 variant and the absence of significant associations for four out of the five investigated SNPs emphasize an important limitation of single-variant prediction. Brace response is unlikely to be determined by one genetic locus, and the predictive value of BNC2 rs10738445 requires validation in independent, ethnically diverse cohorts. These findings therefore support a transition from isolated genetic markers toward multivariable and polygenic predictive approaches.

### 4.4. Polygenic Testing and ScoliScore

Recognition of AIS as a complex polygenic disorder led to the development of multigene approaches intended to estimate the risk of clinically relevant curve progression. One of the best-known examples is ScoliScore, a commercially developed genetic prognostic test combining multiple genetic markers into an individualized risk score [14]. Although originally developed for the prediction of curve progression rather than brace response, the potential relationship between the genetic risk captured by ScoliScore and conservative treatment outcome has also been investigated.

Bohl et al. retrospectively evaluated whether ScoliScore was associated with Providence brace outcome [36]. Genetic samples were obtained from only 16 eligible patients; eight subsequently progressed beyond 45°, whereas eight remained below this threshold. Mean ScoliScore was significantly higher among patients who progressed than among those who did not (176 versus 112; *p* = 0.030) [36].

These findings suggested that polygenic predisposition to progression might contribute to heterogeneity in brace outcomes. However, the very small sample, retrospective design, specific use of Providence bracing, and selected failure threshold substantially limit generalizability. The study should therefore be considered proof-of-concept rather than evidence supporting the routine use of ScoliScore for brace-treatment decisions.

The incremental clinical value of ScoliScore over conventional prognostic assessment has also been investigated. Roye et al. (2012) retrospectively compared ScoliScore-based risk stratification with traditional clinical estimates derived from Cobb angle and Risser sign in 91 patients with AIS [37]. Agreement between the two approaches was limited, with only 25% of patients assigned to the same risk category. ScoliScore classified 36% of patients as low risk and 9% as high risk compared with 2% and 47%, respectively, using conventional clinical estimates. Although these findings suggested that genetic testing may provide information distinct from routinely available clinical parameters, the study did not establish whether such differences improved the prediction of actual curve progression. Therefore, the clinical relevance of ScoliScore should be interpreted cautiously, and its incremental prognostic value requires validation against established clinical models using longitudinal outcomes [37].

Subsequent developments in polygenic prediction have moved beyond fixed commercial panels toward polygenic risk scores (PRS) derived from large-scale genome-wide association data. Otomo et al. developed an AIS PRS using GWAS data comprising 79,211 individuals and demonstrated that polygenic burden could stratify AIS susceptibility and was also associated with Cobb-angle severity [13]. The reported discrimination for AIS susceptibility remained moderate, and incorporation of body mass index with the PRS improved the AUROC from 0.674 to 0.722 [13]. These findings support the biological and predictive potential of genome-wide polygenic information but also illustrate that genetic risk alone is unlikely to provide sufficient discrimination for clinical decision-making.

Importantly, neither ScoliScore nor contemporary AIS PRS should be interpreted as a validated test of brace responsiveness. A score associated with natural-history progression may indirectly correlate with the probability of apparent brace failure because patients with greater underlying progression risk face a more difficult treatment course. Demonstrating a true predictive effect on treatment response, however, requires evidence of interaction between genetic risk and brace exposure, ideally in prospectively characterized cohorts with objective adherence measurements.

More broadly, the evolution from ScoliScore to genome-wide PRS illustrates both the potential and the challenges of translating genetic prediction into clinical practice. Clinical utility depends not only on statistical association, but also on reproducibility across ancestries, calibration in the target population, incremental predictive value beyond established clinical variables, and evidence that use of the score meaningfully improves patient management.

### 4.5. From Individual Variants to Integrated Prediction

Taken together, studies of brace treatment response demonstrate a gradual evolution in genetic prognostic research. Early investigations focused on individual candidate genes selected from hormonal, metabolic, and skeletal pathways, whereas subsequent studies evaluated progression-associated variants and multigene or polygenic risk scores. Associations involving ERα, TPH1, VDR, VDBP, BNC2, and polygenic testing suggest that genetic variation may contribute to heterogeneity in brace outcomes [24,34,35,36]. However, no genetic marker or genetic test currently provides sufficient evidence to replace established clinical predictors or independently guide brace prescription [5,7,10].

An important limitation is that brace outcome is intrinsically multifactorial. Baseline Cobb angle, skeletal maturity, remaining growth, curve characteristics, initial in-brace correction, and treatment adherence are established determinants of outcome [10,31,32,33,38]. Objective monitoring studies have further demonstrated that brace-wear adherence is strongly related to treatment success and that early adherence patterns may predict subsequent brace use [39,40].

These clinical determinants are particularly important when evaluating genetic associations. Failure to account for brace exposure or mechanical correction may create an apparent association between a genetic marker and treatment failure that actually reflects differences in treatment delivery. Conversely, a genetic marker with a modest individual effect could still improve discrimination when incorporated into a properly calibrated multivariable model.

Future predictive strategies should therefore move beyond the search for a single “brace-response gene”. A more clinically relevant objective is the development of integrated models combining genetic or polygenic risk with clinical, radiographic, skeletal-maturity, biomechanical, and objectively measured adherence variables. Contemporary clinical prediction models already demonstrate that meaningful individualized risk estimates can be generated from conventional variables alone [41,42]. Genetic information should therefore be required to demonstrate incremental predictive value beyond these established models before routine implementation can be justified.

Such integrated models could ultimately help estimate the individual probability of progression despite bracing, identify patients requiring closer surveillance, and support more personalized treatment strategies. Their implementation will require prospective multicenter validation, standardized definitions of brace success and failure, objective assessment of brace exposure, ancestry-diverse genetic datasets, and the formal evaluation of calibration and clinical utility.

The principal studies investigating genetic predictors of brace treatment response in AIS are summarized in Table 2.

The studies summarized in Table 2 should not be interpreted as providing equivalent levels of evidence. They differ substantially in sample size, study design, genetic approach, and definition of treatment outcome. In particular, the ScoliScore analysis by Bohl et al. included only 16 genotyped patients and should be regarded as exploratory proof-of-concept evidence. Moreover, radiographic correction, curve progression during treatment, and progression beyond a surgical threshold represent related but distinct outcomes. These methodological differences currently preclude a meaningful quantitative synthesis of the available brace-response studies.

Table 2 also illustrates substantial heterogeneity in treatment delivery and its documentation across genetic studies. Brace type, prescribed daily wear time, adherence assessment, and treatment outcome were not consistently characterized. In particular, objective sensor-based adherence monitoring was generally unavailable or not reported in these genetic studies. Consequently, differences in actual brace exposure may contribute to observed differences between apparent treatment success and failure. This limitation makes it difficult to distinguish a true genotype-specific treatment-response effect from an association driven by variation in treatment delivery, mechanical correction, or adherence.

## 5. From Genetic Markers to Clinical Prediction

### 5.1. Genetic Versus Established Clinical Predictors

The potential clinical value of genetic information in AIS should be considered in the context of established predictors of curve progression and treatment outcome. Age, sex, skeletal maturity, baseline Cobb angle, curve pattern, and remaining growth potential remain central to conventional risk assessment. In particular, combinations of skeletal maturity and curve magnitude provide clinically meaningful estimates of progression risk [2,41].

Contemporary prognostic models demonstrate that substantial predictive information can already be obtained from routinely available clinical and radiographic variables. The BrAIST prognostic model for untreated AIS combined the Simplified Skeletal Maturity System with Cobb angle and curve type and demonstrated strong discrimination for progression to a poor outcome, with c-statistics approaching 0.90 in development and validation cohorts [41]. These findings establish an important benchmark against which molecular predictors should be evaluated.

Genetic markers may nevertheless provide a different and potentially complementary source of prognostic information. Unlike radiographic or maturity-related variables, germline genetic variation is present before clinical progression occurs and remains unchanged throughout follow-up. In principle, genetic information could therefore contribute to risk stratification at an early disease stage, particularly in patients whose conventional clinical indicators provide intermediate or uncertain estimates of risk.

However, genetic associations identified to date have not demonstrated that molecular markers outperform established clinical predictors [4,6]. Most studies of progression- or brace-response-associated variants have been conducted in relatively limited cohorts and have not formally quantified the incremental predictive value obtained by adding genetic information to validated clinical models. Consequently, the relevant clinical question is not whether genetics can replace conventional assessment, but whether it can improve discrimination, calibration, reclassification, or clinical decision-making when added to routinely available variables.

This distinction is essential for future validation studies. A statistically significant genetic association does not necessarily translate into clinically meaningful prediction. Molecular markers should therefore be assessed against strong clinical reference models and should demonstrate measurable incremental benefit before their incorporation into routine risk-stratification algorithms.

### 5.2. Polygenic Risk Scores and Combined Prediction Models

The polygenic architecture of AIS makes prediction based on a single genetic variant biologically unlikely to capture the full inherited component of disease behavior [4,6]. Individual variants generally exert modest effects, and even statistically significant associations may provide limited discrimination at the individual-patient level. This limitation is illustrated by brace-response studies in which only a subset of investigated variants demonstrated significant associations with outcome [24,34,35].

Polygenic approaches provide a conceptually more appropriate framework by combining the effects of numerous variants into a composite estimate of inherited risk. ScoliScore represented an early attempt to translate multigene information into clinical prognostication [14]. However, concerns regarding reproducibility and generalizability emphasized the difficulty of transferring a fixed genetic panel across populations.

More recently, genome-wide polygenic risk scores have provided a broader approach. Otomo et al. developed an AIS PRS using large-scale GWAS datasets and demonstrated an association between increasing polygenic burden and AIS susceptibility as well as curve severity [13]. Although predictive performance remained insufficient for standalone clinical use, these findings indicate that distributed genome-wide genetic information may capture clinically relevant variation more effectively than isolated candidate polymorphisms.

Future AIS PRS should be developed in sufficiently large and ancestrally diverse populations, and importantly, should distinguish between variants associated with disease susceptibility and those specifically associated with curve progression or severity. A PRS developed primarily from case–control susceptibility GWAS cannot automatically be assumed to predict the natural history of an established curve.

Genetic prediction should ultimately be evaluated within combined models. A clinically relevant framework could integrate polygenic risk with baseline Cobb angle, skeletal maturity, growth velocity, curve morphology, family history, and other patient-specific characteristics. Imaging-derived variables may provide an additional layer of information. Three-dimensional spinal parameters measured at the first clinical visit have been incorporated into progression models with encouraging predictive performance [43], while machine-learning approaches using spinal radiographs have demonstrated the feasibility of the automated prediction of future curve progression [44,45,46].

The value of such multimodal approaches lies not in technological complexity itself, but in their ability to capture complementary dimensions of risk. Clinical variables reflect current disease state and remaining growth; imaging features characterize spinal morphology; and genomic information may represent underlying biological predisposition. Integrating these domains may therefore provide more individualized prediction than any single modality alone. Nevertheless, external validation, calibration, interpretability, and demonstration of clinical utility remain essential before such models can influence patient management.

### 5.3. Toward Personalized Brace Treatment

Prediction of brace treatment outcome presents an additional level of complexity because the observed response reflects both the underlying biological tendency of a curve to progress and the mechanical and behavioral characteristics of treatment [10]. A patient with a high intrinsic risk of progression may nevertheless achieve a favorable outcome with effective correction and high adherence, whereas inadequate brace correction or limited wear time may result in progression even in a patient with lower underlying biological risk.

Genetic information should therefore be interpreted as one component of a broader predictive framework rather than as a direct measure of brace effectiveness. Importantly, biological association, prognostic discrimination, and treatment-effect modification represent distinct concepts. An association between a genetic variant and an unfavorable outcome among brace-treated patients does not, by itself, demonstrate that the marker can accurately discriminate prospectively between patients who will succeed or fail treatment. More importantly, such an association does not establish treatment-effect modification, which would require evidence that the effect of bracing differs according to genotype or genetic risk. Demonstrating a true genetic predictor of brace responsiveness therefore requires appropriate characterization of treatment exposure and adjustment for relevant treatment-related and clinical factors, including brace type, prescribed wear time, objectively measured adherence, in-brace correction, skeletal maturity, baseline curve characteristics, and duration of follow-up. Without these data, an observed association with ‘brace failure’ may reflect differences in treatment delivery or underlying progression risk rather than a genotype-specific response to bracing. Associations involving ERα, TPH1, VDR, VDBP, BNC2, and multigene scores suggest that inherited variation may contribute to differences in treatment outcome [24,34,35,36], but these associations must be evaluated alongside skeletal maturity, baseline curve magnitude, curve characteristics, in-brace correction, and brace adherence [10,31,32,33,38,39,40].

Objective adherence data are particularly important in this context. Sensor-based studies have demonstrated substantial interindividual variability in actual brace-wearing time and have shown that greater adherence is associated with more favorable treatment outcomes [39,40,47]. Similarly, early radiographic correction obtained with the brace has repeatedly emerged as an important predictor of subsequent treatment success [38,48]. These factors can therefore confound apparent genetic associations if they are not adequately measured.

This distinction is particularly important when defining “brace failure”. Progression during treatment does not necessarily indicate biological resistance to bracing. Apparent failure may reflect insufficient mechanical correction, inadequate adherence, rapid remaining growth, or combinations of these factors. Genetic studies that fail to account for actual treatment exposure may therefore incorrectly attribute variation in outcome to inherited biological differences.

Future studies should incorporate standardized definitions of brace success and failure, objective monitoring of brace wear, quantitative assessment of initial correction, and validated measures of skeletal maturity. Genetic markers could then be evaluated for their ability to explain residual variation after these established determinants have been considered. Such a design would provide considerably stronger evidence for a true genetic contribution to brace responsiveness.

An integrated clinical-genetic model may ultimately be more useful than attempting to classify patients as genetically “responsive” or “non-responsive” to bracing. For example, BrAIST-Calc has demonstrated the feasibility of estimating the individualized benefit of bracing using conventional clinical variables [41]. Future genomic models should therefore be evaluated according to whether adding genetic information improves such individualized treatment-benefit estimates rather than merely demonstrating an association with treatment outcome.

### 5.4. Toward Personalized Management

The ultimate objective of genetic prognostic research in AIS is not simply to classify patients according to genotype, but to improve individualized clinical decision-making. If sufficiently accurate and externally validated predictive models become available, genetic information could contribute to risk stratification at diagnosis and during conservative treatment.

Patients predicted to have a low probability of clinically significant progression might potentially require less intensive surveillance, whereas those with a high predicted risk could be monitored more closely during periods of rapid growth. In patients meeting conventional indications for bracing, integrated prediction might also help identify individuals at particularly high risk of progression despite treatment, prompting a closer evaluation of brace fit, correction, adherence, and disease evolution.

Importantly, genetic prediction should not be interpreted as genetic determinism. Even a high polygenic risk represents a probability rather than an inevitable clinical outcome. This is particularly relevant in brace-treated patients because treatment itself modifies the natural history of the deformity. Genetic risk should therefore complement rather than override established clinical indications.

The most plausible future framework is one of dynamic multimodal prediction, in which stable genomic information is integrated with variables that change during growth and treatment. Baseline genetic risk could be combined with serial Cobb-angle measurements, skeletal-maturity progression, growth parameters, brace correction, and objectively measured adherence. As new clinical information becomes available, an individual patient’s estimated risk could be updated rather than treated as a fixed value established at diagnosis.

Such an approach would represent a transition from population-level risk factors toward genuinely individualized management. However, its clinical implementation requires prospective multicenter validation, transparent algorithms, demonstration of incremental benefit over conventional assessment, and evidence that risk-guided management improves patient outcomes without generating unnecessary surveillance or treatment.

**Figure 5:**
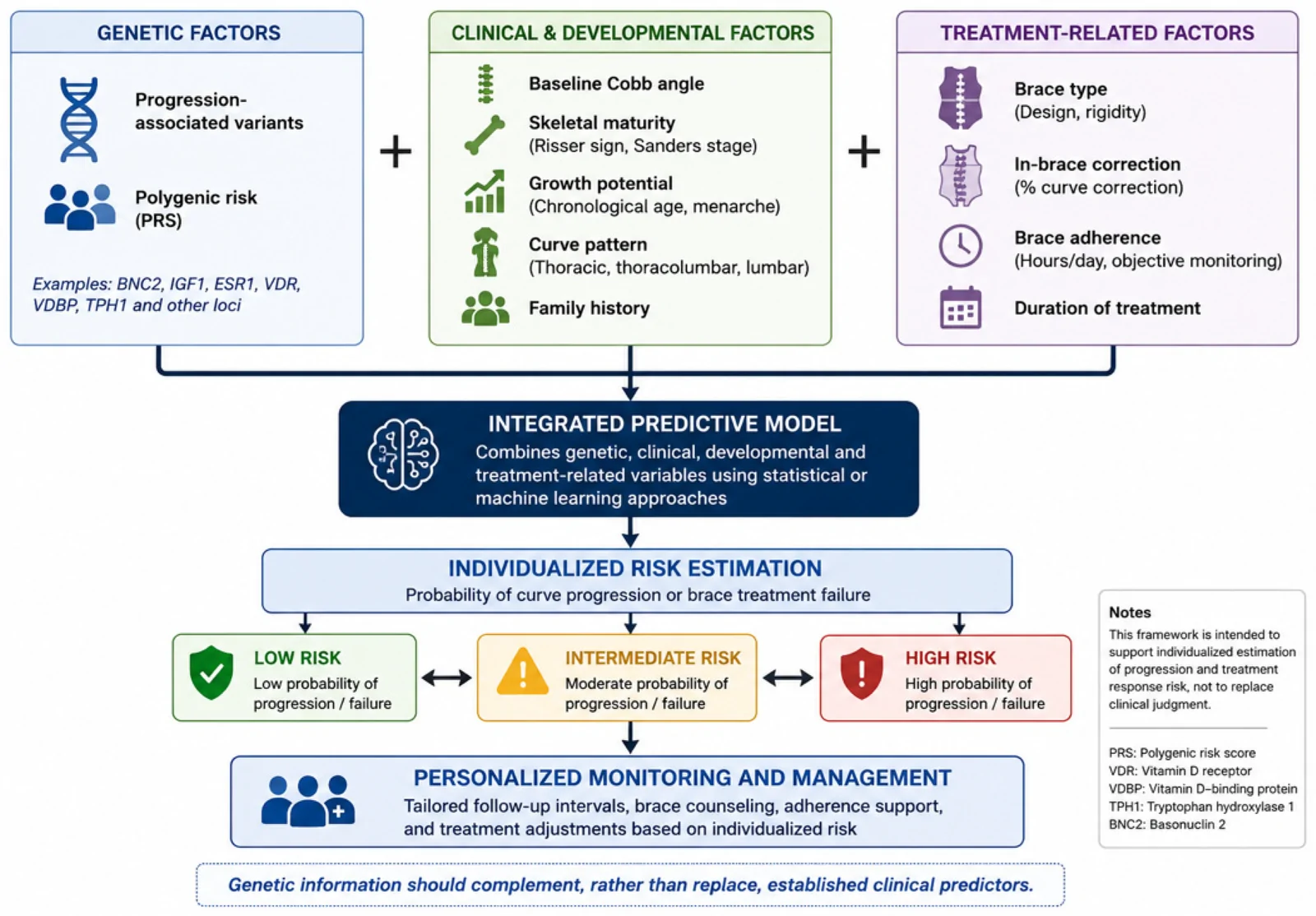
Proposed framework for integrated prediction of curve progression and brace treatment response in adolescent idiopathic scoliosis.

A proposed framework integrating genetic, clinical, developmental, and treatment-related determinants of AIS outcome is illustrated in Figure 5.

### 5.5. Requirements for Clinical Implementation

Before genetic prediction can be incorporated into routine AIS management, several criteria must be fulfilled. First, predictive markers and polygenic scores require independent replication across large and ancestrally diverse populations. Associations identified in a single ancestry group cannot be assumed to provide equivalent predictive performance in other populations because allele frequencies, linkage disequilibrium patterns, and genetic effect sizes may differ [4,5,15].

Second, genetic models must demonstrate clinically meaningful improvement over existing prediction based on conventional variables. Statistical significance alone is insufficient to establish clinical utility. A genetic model should improve discrimination or calibration, meaningfully reclassify patients into clinically relevant risk categories, or improve individualized estimates of treatment benefit. This requirement is particularly important because contemporary clinical, radiographic, and imaging-based models already achieve substantial predictive performance without genetic information [41,42,43,44,45,46].

Third, prospective and external validation is essential. Many existing genetic studies in AIS are retrospective or cross-sectional, whereas clinically useful prediction requires demonstrating that information obtained at the baseline can accurately forecast a future outcome. For brace-response prediction, prospective studies should additionally use standardized treatment protocols, objective adherence monitoring, predefined definitions of treatment success and failure, and sufficiently long follow-up through skeletal maturity [10,39,40].

Fourth, future models should address whether genomic information provides complementary value to increasingly sophisticated imaging and machine-learning approaches. Recent multimodal models combining clinical variables with radiographic information have achieved better performance than single-modality approaches, illustrating the potential value of integrating heterogeneous sources of prognostic information [46,49]. A 2026 multicenter multimodal ensemble model combining frontal and lateral spinal radiographs with clinical variables similarly outperformed individual modalities, although external validation and prospective evaluation remain necessary [49].

Finally, practical and ethical implications of genetic testing in children and adolescents must be considered. Genetic information may have implications beyond the immediate treatment decision, and communication of probabilistic risk requires careful counseling to avoid deterministic interpretation by patients and families. Cost-effectiveness, accessibility, data privacy, equity across ancestry groups, and the psychological consequences of risk classification should therefore be evaluated alongside predictive performance.

At present, available evidence does not support the routine use of genetic testing as an independent determinant of AIS treatment decisions [4,6,24,34,35,36]. Nevertheless, rigorously validated models integrating genomic information with clinical and imaging data may ultimately provide a more comprehensive approach to personalized risk assessment.

## 6. Barriers to Clinical Translation

### 6.1. Limited Sample Sizes and Statistical Power

The ScoliScore analysis of Providence brace outcome illustrates this limitation particularly clearly. Although a significant difference in genetic scores was observed between patients who progressed and those who did not, genetic samples were available for only 16 patients [36]. Such findings are useful for hypothesis generation but cannot establish generalizable predictive performance without replication in substantially larger cohorts.

This limitation becomes even more important for polygenic and multimodal prediction, in which the number of candidate predictors may be large relative to the number of outcome events. Future studies should therefore include adequately powered multicenter cohorts, prespecified analytical strategies, appropriate control of model complexity, and independent validation datasets. Large collaborative datasets will likely be necessary to determine whether genomic information provides reproducible incremental value beyond established clinical predictors.

### 6.2. Population Specificity and Limited Replication

Another major challenge is the population specificity of reported genetic associations. AIS genetic studies have been conducted across Asian, European, and North American populations, and differences in allele frequencies, linkage disequilibrium patterns, genetic background, and environmental exposures may influence reproducibility [5,15,22]. A variant associated with progression or treatment response in one population may therefore have limited predictive value in another.

This issue is particularly important for polygenic risk scores. Predictive models derived predominantly from a single ancestry may demonstrate reduced accuracy when applied to genetically distinct populations. Consequently, successful replication within the discovery population should not be considered sufficient evidence for broad clinical implementation.

Independent validation across multiple ancestry groups is essential. Future prognostic studies should ideally incorporate ancestry-specific analyses, multiethnic cohorts, and external validation to determine whether identified associations represent broadly applicable biological mechanisms or population-specific genetic signals.

### 6.3. Heterogeneous Definitions of Curve Progression and Brace Failure

Comparison between existing studies is further complicated by substantial heterogeneity in outcome definitions. Curve progression may be defined as an increase of 5° or 6°, progression beyond a predefined clinically relevant magnitude, progression to the surgical range, or eventual operative treatment. Similarly, brace success and failure have not been defined uniformly across genetic studies [24,34,36].

This methodological variability may result in substantially different patient groups being classified under the same general outcome. For example, Xu et al. defined brace failure according to progression exceeding 5° or surgery [34], whereas the ScoliScore brace study evaluated progression beyond 45° [36]. Such outcomes represent related but clinically distinct events and should not be assumed to reflect an identical biological phenotype.

Future research should adopt standardized and clinically meaningful outcome definitions whenever possible. Radiographic progression, progression to a clinically important curve magnitude, surgical indication, and failure despite adequately delivered brace treatment should be reported separately. Greater standardization would improve comparison across studies and facilitate future meta-analysis and external validation.

### 6.4. Brace Adherence and Treatment-Related Confounding

Brace-response genetics presents an additional methodological problem because treatment outcome cannot be interpreted independently of treatment exposure. Brace effectiveness is strongly influenced by adherence, yet objective measurements of wear time have not been incorporated consistently into genetic studies [10,39,40,47].

A patient who progresses because of insufficient brace wear may therefore be classified in the same outcome group as a highly adherent patient whose curve progresses despite adequate treatment. These situations are clinically and biologically distinct and may obscure genuine associations between inherited factors and treatment response.

In-brace correction represents another major potential confounder. The mechanical correction achieved by a brace varies substantially among patients and may influence outcome independently of genetic predisposition [38,48]. Differences in brace design, fitting, prescribed daily wear time, and clinical follow-up further increase heterogeneity between studies.

Future genetic studies of brace response should therefore incorporate objective adherence monitoring, standardized measures of initial or in-brace correction, and detailed characterization of treatment exposure. Ideally, genetic predictors should be tested after adjustment for these variables to determine whether they explain residual variation in outcome rather than differences in treatment delivery.

### 6.5. Candidate-Gene Bias and Limited Biological Coverage

Much of the early literature on AIS progression and brace response relied on candidate-gene approaches. Variants were selected because of hypothesized roles in hormonal signaling, skeletal growth, bone metabolism, or other biological pathways considered relevant to scoliosis [4,6]. Although this strategy generated several potentially important associations, it necessarily examined only a small fraction of the biological variation contributing to AIS.

Candidate-gene studies are also particularly vulnerable to selection and publication bias. Positive associations may be more likely to be reported than negative findings, while biologically plausible genes may receive repeated attention despite limited independent evidence. Furthermore, association of a single SNP with a clinical phenotype does not establish either causality or the biological mechanism responsible for the observed outcome.

The transition toward genome-wide, epigenomic, transcriptomic, and other multi-omics approaches may therefore provide a more comprehensive representation of disease biology. Epigenetic markers such as DNA methylation and circulating non-coding RNAs are particularly interesting because, unlike germline variants, they may reflect dynamic biological processes associated with disease evolution [12,19,50,51].

Recent studies of circulating microRNAs have reported signatures associated with AIS severity and progression, while contemporary reviews have highlighted circulating biomarkers as a potentially complementary source of prognostic information [50,51]. However, the current biomarker literature remains heterogeneous, and relatively few markers have been evaluated prospectively for true longitudinal progression.

Multi-omics approaches may ultimately complement germline genetic prediction by integrating stable inherited susceptibility with dynamic molecular signatures. Nevertheless, increasing biological dimensionality also increases analytical complexity, multiple-testing burden, and risk of overfitting. Large longitudinal cohorts and independent validation will therefore remain essential.

### 6.6. Lack of Prospective and External Validation

Association and prediction represent fundamentally different levels of evidence. Demonstrating that the frequency of a genetic variant differs between patients with favorable and unfavorable outcomes does not establish that the variant can accurately predict outcome in an individual patient.

Many currently available AIS genetic studies are retrospective, and relatively few genetic markers have undergone rigorous external validation [4,6]. Even when statistically significant associations have been replicated, studies have rarely demonstrated whether genetic information improves prediction beyond the established clinical variables such as skeletal maturity and baseline curve magnitude.

Contemporary multimodal models already demonstrate promising predictive performance. For example, a large composite model integrating clinical data, global and regional spinal radiographs, and hand radiographs achieved an AUC of 0.84 [45], while more recent multimodal approaches have further highlighted the potential value of combining complementary clinical and imaging information [49].

Prospective longitudinal validation is essential before genetic markers can be considered clinically actionable. Predictive models should be developed and evaluated in independent populations, with prespecified outcomes and appropriate reporting of discrimination, calibration, and clinical utility. Ultimately, the addition of genomic information should demonstrate that it meaningfully improves prediction or management rather than merely increasing model complexity.

### 6.7. Challenges Specific to Polygenic Prediction

Polygenic prediction offers a more biologically plausible approach than reliance on individual variants, but it introduces additional methodological challenges [13]. The selection and weighting of variants strongly influence score performance, and scores developed using susceptibility-associated loci may not necessarily predict curve progression or treatment response. Disease susceptibility, progression, severity, and response to treatment represent related but distinct phenotypes and should not be assumed to share identical genetic determinants.

PRS performance may also vary according to ancestry, age, sex, phenotype definition, and the characteristics of the GWAS from which variant weights were derived. A score optimized to distinguish individuals with AIS from unaffected controls may perform poorly when asked to distinguish progressive from stable curves among patients who already have AIS.

Future progression-specific PRS should therefore ideally be derived from large longitudinal cohorts with standardized phenotyping. Their performance should subsequently be evaluated independently and compared with established clinical prediction models. Similar principles apply to brace-response scores, which require the accurate characterization of treatment exposure and adherence.

Ultimately, polygenic prediction should be viewed as one layer within a broader multimodal framework rather than as an isolated solution to AIS prognostication.

### 6.8. Ethical and Practical Considerations

Genetic testing in children and adolescents also raises practical and ethical considerations that extend beyond predictive accuracy. Genetic risk estimates are probabilistic and may be difficult for patients and families to interpret, particularly when the available evidence does not support a definitive prediction of disease progression or treatment outcome.

Premature use of poorly validated genetic information could lead to unnecessary anxiety, false reassurance, or inappropriate modification of treatment. These concerns are especially relevant in AIS because effective clinical monitoring and conservative treatment options already exist, and treatment decisions may have long-term consequences during a critical developmental period.

Communication of genetic risk in children and adolescents requires particular caution because probabilistic results may be misunderstood as deterministic predictions. Being classified as carrying a ‘high-risk’ genotype could generate anxiety, alter the child’s or family’s perception of future disease progression, or create unrealistic expectations regarding treatment outcome. Genetic counseling should therefore accompany clinically implemented genetic testing to explain the probabilistic nature, limitations, and uncertainty of risk estimates and to support informed interpretation by patients and families [52,53].

Because patients with AIS are predominantly minors, consent procedures should also be developmentally appropriate. Parental permission should be accompanied, whenever appropriate, by the child or adolescent’s informed assent, with information presented in language suitable for age and level of understanding [52,53]. Particular caution is required if a genetic result is proposed as a basis for modifying surveillance intervals, brace treatment, or other aspects of management. Such changes should only be considered when the genetic information has demonstrated sufficient clinical validity and utility, and should be incorporated into shared decision-making rather than used as an isolated determinant of care.

Data privacy, informed consent, accessibility, and potential disparities in genetic testing should also be considered. If future genetic prediction tools are developed predominantly in selected populations or are available only in specialized settings, their implementation could widen rather than reduce disparities in care.

Clinical translation therefore requires not only statistical and biological validation, but also evidence that genetic testing is understandable, accessible, cost-effective, and capable of improving patient-centered outcomes without introducing disproportionate harms.

### 6.9. Current State of Evidence

Taken together, the available evidence supports a contribution of genetic and epigenetic variation to AIS curve progression and suggests a potential influence on brace treatment outcome. Nevertheless, substantial methodological limitations continue to prevent immediate clinical translation. Most reported associations remain insufficiently replicated across independent and ancestrally diverse populations, effect sizes are generally modest, and evidence that molecular information improves prediction beyond established clinical variables remains limited [4,6,13,25].

Genetic testing should therefore currently be regarded as a research and prognostic-development tool rather than a standalone determinant of clinical management. Similarly, emerging epigenetic and circulating biomarkers should be considered promising investigational tools rather than clinically validated predictors [12,50,51].

The most promising direction is not the identification of a single progression or brace-response variant, but the development of rigorously validated models integrating polygenic and molecular information with clinical, radiographic, developmental, biomechanical, and treatment-related factors. Such integrated approaches provide the conceptual basis for the future research priorities discussed below.

## 7. Future Directions

### 7.1. Prospective Multicenter Cohorts and Standardized Phenotyping

Future research on genetic prediction in AIS should prioritize large, prospective, multicenter cohorts with standardized clinical, radiographic, and molecular characterization. Most currently available studies were conducted in relatively limited populations, frequently using retrospective designs and heterogeneous definitions of progression or treatment failure. These limitations reduce statistical power and complicate comparison and replication across studies [4,6].

Prospective studies should define clinically meaningful outcomes before patient recruitment and follow participants throughout the period of skeletal growth. Baseline characteristics should include age, sex, curve magnitude and pattern, skeletal maturity, growth potential, and family history, together with longitudinal measurements of curve behavior. For brace-treated patients, treatment characteristics should additionally include brace type, prescribed wear time, objectively measured adherence, in-brace correction, and treatment duration [10,39,40].

Importantly, future biomarker studies should distinguish cross-sectional associations with curve severity from true longitudinal prediction of progression. A recent systematic review of circulating and tissue biomarkers identified 29 studies involving more than 4000 participants but concluded that relatively few evaluated longitudinal progression and that predictive evidence remains limited [54].

Harmonization of phenotypes and outcome definitions across centers would facilitate pooled genomic analyses and external validation. International collaboration will be particularly important because sufficiently powered genomic and polygenic studies require substantially larger cohorts than most individual institutions can provide.

### 7.2. From Candidate Genes to Genome-Wide and Polygenic Approaches

Future studies should progressively move beyond isolated candidate–gene associations toward genome-wide and sequencing-based approaches. Candidate-gene studies have provided important initial evidence that genetic variation may influence progression and brace outcome, but they interrogate only a limited proportion of the genetic architecture underlying these complex phenotypes [4,6].

Genome-wide association studies specifically designed around longitudinal progression phenotypes could identify loci that differ from those primarily associated with AIS susceptibility. Existing findings involving MIR4300HG and more recent severity-associated loci such as GAK and KLF12 illustrate the potential of phenotype-specific genome-wide approaches [16,21,25]. However, prospective longitudinal phenotyping remains necessary to distinguish genetic determinants of true progression from markers associated primarily with final curve severity.

Sequencing approaches may additionally reveal rare variants with larger effects in selected patients or families with particularly aggressive disease. Importantly, susceptibility, curve progression, final severity, and treatment response should be analyzed as distinct but potentially overlapping phenotypes rather than being treated as interchangeable genetic outcomes.

The accumulation of sufficiently large genomic datasets could enable the development of progression-specific polygenic risk scores. Unlike scores derived predominantly from susceptibility-associated variants, these models should be trained directly against clinically meaningful longitudinal outcomes and validated independently across different ancestry groups before clinical interpretation [13].

### 7.3. Integration of Genetic and Clinical Predictors

Genetic information is unlikely to achieve optimal predictive performance when considered in isolation. Future models should therefore integrate genomic data with established clinical and radiographic predictors, including baseline Cobb angle, skeletal maturity, growth potential, curve pattern, and longitudinal changes during follow-up [2,41,42,43].

For brace-treated patients, treatment-related variables are particularly important. Objective measurements of brace adherence and quantitative assessment of in-brace correction should be incorporated into predictive models to distinguish biological progression despite adequate treatment from progression associated with insufficient treatment exposure or mechanical correction [38,39,40,47,48].

The incremental contribution of genetic information should be evaluated against well-performing models based exclusively on routinely available clinical and radiographic variables. A combined clinical-genetic model should not be considered superior simply because a genetic marker is statistically associated with outcome. Rather, the addition of genetic information should demonstrate measurable improvement in predictive performance beyond the clinical model alone. This should include an assessment of discrimination, calibration, and incremental predictive value, followed by independent external validation in populations distinct from those used for model development. Ultimately, clinical utility should also be demonstrated by showing that the additional genetic information meaningfully improves risk stratification or clinical decision-making, rather than merely increasing model complexity. For brace-treated patients, this comparison is particularly important because conventional clinical and treatment-related variables—including baseline Cobb angle, skeletal maturity, growth potential, curve pattern, brace type, in-brace correction, prescribed wear time, and objectively measured adherence—already provide substantial prognostic information. Genetic data would therefore be clinically relevant only if they provide reproducible information beyond these established predictors. Appropriate evaluation should compare the performance of a clinical model with that of the same model after the addition of genetic variables, using prespecified outcomes and independent validation cohorts.

Contemporary machine-learning evidence reinforces this principle. A 2024 systematic review of 15 studies evaluating machine-learning approaches for AIS progression found considerable promise but also highlighted problems involving interpretability, heterogeneous data, and the need for larger multicenter studies [55]. Future genetic models should therefore be developed within rigorous predictive frameworks rather than simply adding genomic variables to increasingly complex algorithms.

### 7.4. Multi-Omics and Functional Characterization

Genomic variation represents only one component of the biological processes underlying AIS progression. Integration of genomics with transcriptomic, epigenomic, proteomic, metabolomic, and circulating biomarker data may provide a more comprehensive understanding of why apparently similar spinal deformities follow different clinical trajectories.

Epigenetic information is particularly relevant because it may provide a biological link between inherited predisposition, growth-related influences, and dynamic disease behavior. DNA methylation studies have identified candidate progression-associated signals [12,19], while recent investigations of LBX1, ESR1, and ESR2 methylation further illustrate the ongoing evaluation of gene-specific epigenetic alterations in idiopathic scoliosis, although associations with disease severity have not been consistently demonstrated [56].

Circulating non-coding RNAs represent another promising direction. Genome-wide profiling of circulating microRNAs in patients with AIS identified a six-miRNA panel associated with the subsequent development of severe scoliosis [50]. The reported model demonstrated extremely high classification performance; however, such results require rigorous independent external validation before they can be considered clinically applicable.

Longitudinal molecular profiling may ultimately be more informative than single-time-point biomarker assessment. Recent biomarker reviews have emphasized that repeated sampling during growth could help determine whether molecular changes precede curve progression, occur as a consequence of increasing deformity, or simply correlate with disease severity [51,54].

Multi-omics approaches could also bridge the gap between statistical association and biological mechanism. Variants associated with progression or brace outcome should be investigated for effects on gene expression, regulatory activity, cellular function, and relevant musculoskeletal pathways. Such functional characterization is essential because statistical association alone does not establish causality.

### 7.5. Artificial Intelligence and Multimodal Prediction

The increasing availability of genomic, radiographic, biomechanical, surface-topography, and longitudinal clinical data creates opportunities for multimodal predictive modeling. Machine-learning approaches may be particularly useful for identifying complex nonlinear interactions between genetic and conventional prognostic variables that are difficult to capture using simpler statistical models.

AI applications in AIS have expanded rapidly. A recent scoping review identified 40 studies applying artificial intelligence to AIS, with prediction representing one of the major areas of investigation; however, limitations related to external validation, transparency, and clinical implementation remain common [57].

Recent progression models illustrate both the potential and the limitations of this approach. Multimodal models integrating clinical and imaging features have demonstrated promising discriminatory performance [46,49], while Kubota et al. developed machine-learning regression models in 201 female patients with mild-to-moderate AIS, reporting R^2^ values of 0.73 for the prediction of Cobb angle at one year and at the final visit [58].

Non-radiographic information may also become relevant. A 2026 prospective multicenter study investigated surface-topography parameters and angle of trunk rotation within a random-forest model for short-term Cobb-angle progression [59]. Although the cohort was small and the detection of true progression remained less accurate than the identification of stable curves, the study illustrates the possibility of incorporating radiation-free monitoring data into future dynamic prediction systems.

A future AIS model could therefore integrate polygenic risk, molecular biomarkers, skeletal maturity, three-dimensional curve characteristics, growth parameters, family history, brace correction, adherence, and serial non-radiographic measurements. Risk estimates could be updated as new information becomes available during growth and treatment rather than remaining fixed at diagnosis.

However, increasingly complex models do not automatically provide greater clinical utility. AI-based approaches require transparent reporting, appropriate handling of missing data and population heterogeneity, external validation, calibration assessment, and comparison with simpler prediction models [55,57]. Explainability will also be important if model outputs are expected to influence decisions involving children and adolescents.

### 7.6. Development of Clinically Actionable Prediction Models

The ultimate goal of prognostic research should be the development of models that improve clinical decisions rather than the prediction accuracy alone. Future studies should therefore define in advance how a predicted risk estimate could alter patient management.

Validated individualized risk estimates might eventually contribute to decisions regarding the intensity of surveillance, timing of follow-up, or identification of patients requiring particularly close monitoring during periods of rapid growth. In brace-treated patients, prediction of a high residual risk of progression despite adequate treatment might prompt an earlier reassessment of brace fit, in-brace correction, adherence, and overall treatment strategy.

This objective is distinct from merely achieving a high AUC. Models should demonstrate appropriate calibration, clinical usefulness across relevant decision thresholds, and preferably incremental benefit over established clinical prediction tools. Decision-curve analysis and prospective impact studies may be useful for determining whether improved statistical prediction translates into improved management.

Genetic risk should not be used in isolation to deny bracing or accelerate invasive treatment. Clinical implementation would require evidence that model-guided management improves meaningful patient outcomes compared with current practice. Prospective decision-impact studies, and ultimately, interventional trials, may therefore be required in addition to conventional predictive validation.

### 7.7. Proposed Roadmap Toward Clinical Translation

Based on the current evidence, the translation of genetic prediction into AIS management can be conceptualized as a sequential process:

Progression, severity, and treatment-response phenotypes should first be clearly distinguished, standardized, and investigated in sufficiently large prospective multicenter cohorts.

Robust genetic, epigenetic, or other molecular associations should be independently replicated across diverse populations.

Validated biomarkers should be incorporated into multivariable models together with established clinical, radiographic, developmental, and treatment-related predictors.

Integrated models should undergo independent external validation and demonstrate meaningful incremental predictive value, calibration, and clinical utility compared with established non-genetic models.

Prospective impact studies should determine whether model-guided risk stratification actually improves clinical decision-making and patient outcomes.

Only after these steps have been completed should genetic prediction be considered for routine clinical implementation. This staged approach would reduce the risk of the premature adoption of biomarkers based primarily on statistical association and help ensure that future precision-medicine tools address clinically relevant questions.

## 8. Conclusions

Genetic research in adolescent idiopathic scoliosis has progressively evolved from the identification of susceptibility loci toward the prediction of clinically relevant outcomes. Available evidence indicates that inherited variation contributes to AIS susceptibility and may also influence curve severity, progression, and response to conservative treatment. Candidate-gene studies have reported associations involving hormonal, metabolic, and developmental pathways, while genome-wide investigations have identified susceptibility- and phenotype-associated loci including BNC2, MIR4300HG, GAK, and KLF12. Studies of brace outcome have additionally suggested potential associations involving ERα, TPH1, VDR, VDBP, and BNC2, although these findings remain insufficient for direct clinical application.

The available evidence also demonstrates an important distinction between disease susceptibility, curve severity, longitudinal progression, and treatment response. These phenotypes are related but not interchangeable, and genetic markers identified for one outcome should not automatically be assumed to predict another. This distinction is particularly important for brace-response research, in which observed progression reflects not only intrinsic biological risk, but also skeletal maturity, baseline deformity, mechanical correction, and treatment adherence.

No currently available genetic marker, polygenic score, or molecular biomarker has demonstrated sufficient independent validation and incremental predictive value to replace established clinical assessment. Earlier approaches such as ScoliScore illustrated the potential of multigene prediction but also highlighted challenges related to population specificity and generalizability. Contemporary polygenic risk scores provide a more comprehensive representation of inherited susceptibility, yet progression-specific and treatment-response-specific scores remain to be adequately developed and validated.

Emerging epigenetic and circulating biomarkers may provide additional information beyond static germline variation. DNA methylation signatures and circulating microRNAs are particularly promising because they may capture biological processes associated with active disease evolution. However, most biomarker evidence currently relates to disease severity rather than prospectively observed progression, and recent systematic evidence confirms that longitudinal predictive validation remains limited.

The future clinical value of genetics in AIS is therefore likely to depend on integration rather than substitution. Large prospective multicenter cohorts, standardized longitudinal phenotyping, ancestry-diverse genomic datasets, objective characterization of brace exposure, and independent external validation will be essential. Genetic and molecular information should ultimately be evaluated alongside established clinical predictors, skeletal maturity, radiographic and three-dimensional imaging characteristics, growth-related variables, biomechanical factors, and objective adherence data.

Multimodal prediction represents a particularly promising direction toward personalized AIS management. Rather than searching for a single “progression gene” or “brace-response gene”, future research should determine whether genomic and molecular information provides meaningful incremental value over increasingly accurate clinical and imaging-based prediction models. Only models that demonstrate robust external validity, appropriate calibration, clinical utility, and an ability to improve patient management should progress toward implementation.

At present, genetic testing should therefore be regarded as a promising component of research and future precision management in AIS rather than an independent determinant of surveillance, brace prescription, or surgical decision-making. The transition from genetic association to clinically actionable prediction will depend on rigorous validation and demonstration that integrated risk assessment improves outcomes for individual patients.

## Figures and Tables

**Table 1:** Primary genetic and epigenetic studies evaluating AIS curve progression or curve severity, with interpretation according to study design and strength of evidence.

Study	Study Design/Population	Gene/Marker	Outcome Phenotype	Main Finding	Replication/Evidence Interpretation
Moon et al., 2013 [17]	Candidate-gene study; Korean AIS population	IGF1, LAPTM4B; selected SNPs	Curve severity/clinical phenotype	Selected polymorphisms were associated with AIS phenotype, including curve severity	Exploratory candidate-gene evidence; population-specific findings and limited independent validation preclude interpretation as an established progression predictor
Jiang et al., 2013 [29]	Candidate-gene study; Chinese AIS cohort	LBX1 rs11190870	Cross-sectional curve severity (Cobb angle)	The TT genotype was associated with greater mean Cobb angle than the CC genotype	Initial positive severity association; subsequent studies produced inconsistent results. Cross-sectional severity should not be interpreted as longitudinal progression
Ogura et al., 2017 [16]	Genome-wide association and replication study; 2543 patients with AIS	MIR4300HG rs35333564	Curve progression	rs35333564 was associated with progression; functional experiments demonstrated reduced enhancer activity of the risk allele	Comparatively strong progression-focused evidence based on large GWAS and replication cohorts; subsequent Chinese evidence provided additional cross-population support, although broader validation remains necessary
Mao et al., 2018 [19]	Epigenetic association study; AIS patients stratified by curve severity	COMP promoter methylation	Cobb angle/curve severity	Increased COMP promoter methylation was associated with reduced expression and greater curve severity	Supports an epigenetic association with curve phenotype; primarily severity-associated rather than prospective longitudinal progression evidence
Meng et al., 2018 [12]	Epigenome-wide discovery in monozygotic twins discordant for progression, followed by independent validation	cg01374129 DNA methylation	Progressive vs. non-progressive AIS	Hypomethylation of cg01374129 was independently associated with progression; reported AUC 0.827	Promising progression-focused epigenetic evidence with validation, but further independent external validation is required before clinical implementation
Wright et al., 2025 [18]	Retrospective candidate-gene study; 72 patients with longitudinal AIS follow-up	COMT rs4680 and MTHFR C677T/A1298C combinations	≥20° Cobb progression or spinal surgery	Progression occurred in 78.0% of patients with combined low-activity genotypes versus 48.4% of the comparison group	Exploratory, hypothesis-generating evidence; very small cohort, selected genotype groups, retrospective design, and absence of external validation substantially limit generalizability
Dai et al., 2026 [25]	Genome-wide discovery and replication cohorts comparing female patients with severe and mild AIS	GAK rs2061846; KLF12 rs7330031	Severe (>40°) vs. mild (<30°) curves	Both loci were replicated as severity-associated; KLF12 expression was negatively correlated with Cobb angle	Replicated genome-wide evidence for curve severity, but the cross-sectional severe-versus-mild phenotype is not equivalent to prospective longitudinal progression

**Table 2:** Primary studies evaluating genetic predictors of brace-treatment outcomes in adolescent idiopathic scoliosis, with interpretation according to study design and strength of evidence.

Study	Study Design/Population	Genetic Marker/Test	Brace Type and Prescribed Regimen	Adherence Assessment	Brace-Treatment Outcome	Main Finding	Evidence Interpretation/Major Limitations
Xu et al., 2011 [34]	Retrospective candidate-gene study; 312 patients with AIS treated with bracing	ESR1, ESR2, TPH1, MTNR1B, MATN1; including ESR1 rs9340799 and TPH1 rs10488682	Milwaukee or Boston brace according to curve pattern; initially 22 h/day, subsequently adjusted according to curve evolution and skeletal maturity	Patient/parent-reported actual daily wear time; actual-to-prescribed wear ratio calculated; only patients with >75% compliance included	Brace failure defined as progression >5° or progression requiring surgery	ESR1 rs9340799 and TPH1 rs10488682 were independently associated with brace effectiveness	Relatively large candidate-gene cohort, but population-specific findings and limited independent replication restrict generalizability
Wang et al., 2016 [35]	Candidate-gene study; AIS patients undergoing brace treatment	VDR BsmI; VDBP rs222020	Standardized brace-wearing plan; brace selected according to scoliosis type; initially 22 h/day, subsequently adjusted according to treatment response and skeletal maturity; follow-up 6–36 months	Self-reported actual daily wear time at 3-month follow-up visits; patients with <75% compliance were excluded; no objective sensor-based monitoring reported	Success: Cobb-angle increase ≤5°; failure: increase >5° or discontinuation of bracing for surgery	VDR BsmI Bb and VDBP rs222020 CC genotypes were associated with poorer correction of several radiographic parameters	Population-specific candidate-gene evidence with limited external validation; radiographic correction is not equivalent to longitudinal brace failure
Bohl et al., 2016 [36]	Retrospective proof-of-concept study; 16 genotyped patients treated with a Providence brace	ScoliScore multigene prognostic score	Providence nighttime brace; prescribed wear duration not reported in the accessible study information	Objective adherence monitoring not reported in the accessible study information	Progression to >45°	Mean ScoliScore was higher among patients who progressed than among those who remained below the threshold (176 vs. 112; *p* = 0.030)	Exploratory evidence only; extremely small sample, retrospective design, specific brace type, and selected outcome threshold preclude clinical generalization
Dai et al., 2025 [24]	Genetic association study; 259 female patients with AIS undergoing brace treatment	BNC2 rs10738445; SOX9/KCNJ2 rs12946942; TBX1 rs1978060; CHD7 rs1017861; MIR4300HG rs35333564	Not specified in the publicly available report; patients were described as ‘well-braced	Patients were described as ‘well-braced’; the method used to quantify adherence could not be determined from the publicly available report.	Brace failure/curve progression	BNC2 rs10738445 C allele was associated with increased risk of brace failure (OR 1.59); the other four variants were not significantly associated with outcome	Larger contemporary cohort and inclusion of negative findings strengthen interpretation, but independent prospective and multiethnic validation is required

## Data Availability

No new data were created or analyzed in this study. Data sharing is not applicable to this article.

## Abbreviations

AI	Artificial intelligence
AIS	Adolescent idiopathic scoliosis
AUC	Area under the curve
BMI	Body mass index
BNC2	Basonuclin 2
CI	Confidence interval
COMT	Catechol-O-methyltransferase
DNA	Deoxyribonucleic acid
ERα	Estrogen receptor alpha
ERβ	Estrogen receptor beta
GWAS	Genome-wide association study
IGF1	Insulin-like growth factor 1
MATN1	Matrilin 1
ML	Machine learning
MTHFR	Methylenetetrahydrofolate reductase
MTNR1B	Melatonin receptor 1B
OR	Odds ratio
PRS	Polygenic risk score
SNP	Single-nucleotide polymorphism
TPH1	Tryptophan hydroxylase 1
VDBP	Vitamin D-binding protein
VDR	Vitamin D receptor
